# Pyrolysis Behavior, Kinetic Analysis, and Biochar Production from Waste Flowers

**DOI:** 10.1021/acsomega.4c10700

**Published:** 2025-02-18

**Authors:** Richa Gupta, Ranjeet Kumar Mishra, Kaustubha Mohanty

**Affiliations:** 1 Department of Chemical Engineering, Indian Institute of Technology Guwahati, Guwahati, Assam 781039, India; 2 Department of Chemical Engineering, Manipal Institute of Technology, Manipal Academy of Higher Education, Manipal, Karnataka 576104, India

## Abstract

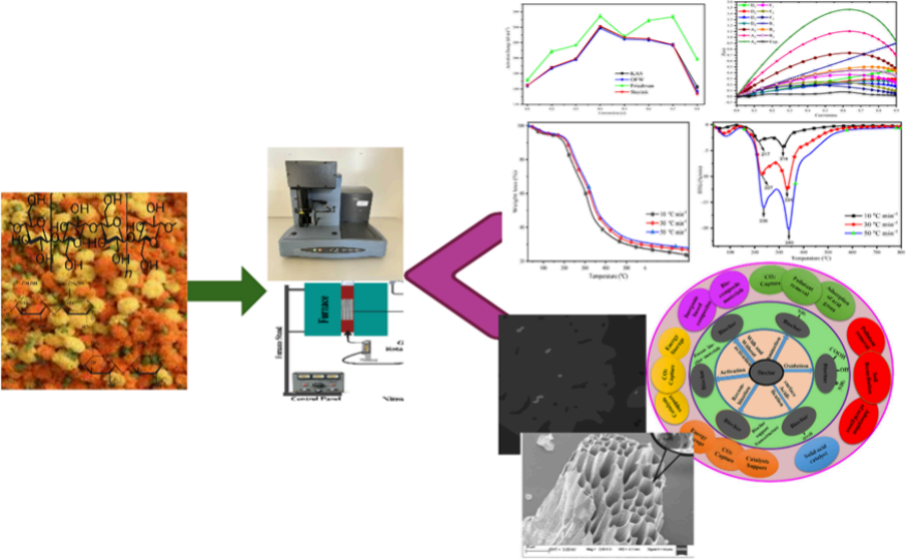

Waste flowers constitute a significant portion of organic waste, offering the potential for sustainable waste management through pyrolysis. This study explores the pyrolysis behavior, kinetic parameters, and biochar production from waste flowers. Thermogravimetric analysis (TGA) was employed to examine thermal degradation characteristics under varying heating rates (10, 20, and 50 °C min^–1^). Kinetic analysis was performed using model-free methods such as the Friedman method (FM), Ozawa–Flynn–Wall (OFW), Starink method (STM), Kissinger–Akahira–Sunose (KAS), and Criado model to determine the pyrolysis kinetic parameters. Further, the biochar was produced in a semibatch reactor at 450 °C with a 10 °C min^–1^ heating rate and 100 mL min^–1^ nitrogen flow rate. The characterization of the biochar included proximate and elemental analysis, calorific value, bulk density, Brunauer–Emmett–Teller (BET) surface area, pH, Fourier transform infrared spectroscopy (FTIR), field emission scanning electron microscopy (FESEM), energy-dispersive X-ray (EDX) analysis, and water-holding capacity. The decomposition results were confirmed in three stages: moisture removal, active pyrolysis, and residue formation. Kinetic results revealed a multistep reaction mechanism, with average activation energies of 236.35, 232.29, 234.74, and 221.50 kJ mol^–1^ derived from KAS, OFW, STM, and FM, respectively. Pyrolysis of marigold flowers (MG) yielded 36.64 wt % biochar at 450 and 10 °C min^–1^ heating rate. Further, the biochar exhibited a 57.10% carbon content, 33.57 MJ kg^–1^ higher heating value (HHV), 9.96 m^2^ g^–1^ BET surface area, and 29.14 mV zeta potential, demonstrating its potential for soil amendment, carbon sequestration, and pollutant adsorption. This study emphasizes the value of MG as a feedstock for biochar production, contributing to circular economy initiatives.

## 1 Introduction

The shortage of fossil fuel due to its depleting resources, increasing environmental impacts, and political commitment has led to great interest in research and developments in alternative energy sources. The UN climate panel has targeted a 50–80% reduction in greenhouse gases by 2050.^1^ Reducing the dependency on fossil fuels and shifting toward renewable fuels are essential to achieve this target. Biomass has drawn the most attention of all the energy sources due to its advantages to the environment. Biomass refers to plant matter, which is abundantly available (220 billion tons annually) and a low-cost renewable energy source.^2^ Studies testified that roughly 10–14% of the world’s energy is produced by biomass.^3^ Further, the grown biomass and waste (from harvesting and postprocessing) are regarded as appealing sources for the generation of fuel and energy. The primary suppliers of lignocellulosic biomass are aquatic plants, forestry waste, crop residue from agriculture, and other energy crops.^4^ The residue obtained after processing and harvesting requires proper disposal. Furthermore, utilizing waste byproducts for fuel and energy production minimizes waste generation and land use while enhancing economic profitability through complete resource utilization. This approach also supports the advancement of a biobased economy. Moreover, efficient biomass utilization for energy production contributes to job creation and economic stability.

The floral waste sector is witnessing significant growth and offers diverse benefits in India. This sector not only generates meaningful employment but also helps divert biodegradable waste from landfills, supporting environmental conservation. Floral waste, primarily sourced from spiritual sites, often ends up in landfills or water bodies, posing health risks and damaging aquatic ecosystems. A UN Climate Change report notes that the Ganga River alone receives over 8 million metric tons (MMT) of flower waste annually.^5^ However, this waste can also be used to produce value-added chemicals and biochar. *Tagetes* is a genus within the Asteraceae family comprising around 50 species of annual and perennial herbaceous plants. Known commonly as marigolds, these plants are recognized for their vibrant blooms and are widely cultivated for their ornamental and medicinal value.

The two subsequent stages for turning biomass into renewable fuel are thermochemical methods (TCMs) and biochemical methods (BCMs). The thermochemical method breaks up biomass in minutes or seconds, whereas the biochemical method takes longer to convert biomass. The main thermochemical processes include pyrolysis, combustion, gasification, and hydrothermal liquefaction. The thermal cracking of organic matter or biochar at moderate temperature (400–900 °C) in the privation of oxygen or air atmosphere is known as pyrolysis.^6^ Among the various thermochemical processes, pyrolysis stands out for its ability to convert materials into solid, liquid, and syngas products. Recently, it has gained prominence as a more efficient and versatile conversion method, particularly for the pyrolysis of waste plastics and biomass, including their copyrolysis.^7^

Kinetic modeling is necessary for precise predictions of biomass degradation behavior under various conditions. Biomass pyrolysis is a multifaceted process involving numerous reactions occurring within seconds or minutes, heavily influenced by the operational conditions. This inherent complexity has led to extensive literature exploration of various theories to explain the mechanisms behind biomass breakdown during pyrolysis. TGA has been shown to be a valuable and efficient paralyzer in revealing the biomass pyrolysis process and kinetics. Two primary models were used in the TGA-based analysis using TGA data: the nonisothermal and the isothermal. Since the isothermal process requires holding time and rates, nonisothermal methods typically exhibit a smaller range of inaccuracies compared to isothermal approaches.^8^ In contrast, nonisothermal methods are derived from less complex studies or experimental procedures. They allow for continuous kinetic estimation across the entire temperature range, reducing potential errors associated with thermochemical induction techniques. Nonisothermal models can be divided into two types: model-free and model-fitting. Model-free approaches are preferred due to their minimal error. As higher heating rates can introduce disorder due to longer residence times, nonisothermal methods are considered the most effective for estimating pyrolysis reaction kinetics, mainly when applied at lower and variable heating rates.^9^ Additionally, shorter intervals were preferred over longer intervals, as they yielded more accurate results during TGA pyrolysis by shifting the peaks. The activation energy is determined using the iso-conversional model, such as Kissinger–Akahira–Sunose (KAS), Friedman method (FM), Ozawa–Flynn–Wall (OFW), Starink method (STM), and Criado model. These models were chosen as they are well-established, model-free approaches that offer reliable results in determining the activation energy (*E*_a_) and other kinetic parameters without requiring assumptions about the reaction mechanism. The KAS and OFW models are widely used for analyzing nonisothermal decomposition processes and provide accurate activation energy estimations at different conversion levels. The FM method is effective for analyzing pyrolysis kinetics across a broad range of heating rates. In contrast, the STM method is known for its precision in determining the activation energy and elucidating reaction mechanisms in complex systems. The Criado model is particularly valuable for analyzing multistep reactions and providing reliable results in nonlinear temperature conditions. These models essentially operated under the assumptions of a continuous heating process and activation energy. Temperature was the primary function in determining the chemical and physical characteristics of biomass and kinetic analysis.^10^ TGA methodologies have been widely used by researchers to explain the reaction kinetics of pyrolysis, often using single-step reaction models. Nonetheless, these models fail to accurately forecast the effects of operational factors on the product yield. The specific kinetics of the generation of tar, gases, and biochar can be explained by two-step or parallel reaction models. The three-step model’s multipseudo feature was utilized to clarify the intermediate products that were obtained from the primary breakdown.^11^ Moreover, the kinetic properties of this highly complex and adaptable process were estimated using a single-step or one-step reaction kinetics model. Di Blasi (2008) employed three parallel reactions to establish the pyrolysis of biomass, while the three independent models were utilized to clarify the kinetics of the pyrolysis reaction. Furthermore, the kinetic equation (random nucleation, nucleation, growth, diffusion, and phase boundary) was proposed by Criado et al. (1989) to explain solid processes.^12^ Regrettably, these models lack accurate physical implications and, more importantly, fail to ensure their applicability. Also, it was noted that MG was utilized for neither biochar production at 450 °C and 10 °C min^–1^ heating rate nor kinetic analysis using model-free methods (KAS, OFW, STM, and FM) before to the best knowledge of the authors. Also, it is essential to elucidate the kinetic decomposition mechanism of biomass, which offers a comprehensive understanding of the degradation process. The iso-conversional models, which do not rely on assumptions, are considered the most consistent methods for determining the activation energy (*E*_a_) and frequency factor (*n*).^8^

Although numerous studies have investigated the kinetics and pyrolysis behavior of biomass, no data are currently available to estimate the pyrolysis characteristics of MG and the biochar formation using these techniques. Therefore, this study aims to explore the physicochemical properties, thermal degradation behavior, and biochar production. To understand its kinetic decomposition mechanism, five model-free methods, i.e., Kissinger–Akahira–Sunose (KAS), Friedman method (FM), Ozawa–Flynn–Wall (OFW), Starink method (STM), and Criado model, were employed at three different heating rates (10, 30, and 50 °C min^–1^). The behavior of biomass degradation was also explored using thermodynamic analysis. A semibatch reactor was used to generate the biochar at 450 and 10 °C min^–1^ heating rate, and its chemical and physical characteristics were assessed using proximate and elemental analysis, HHV, bulk density, BET surface area, pH, water holding capacity (WHC), FTIR, SEM, and EDX.

## 2 Material and Methods

### 2.1 Sample Collection and Preparation

Marigold flowers (*Tagetes erecta*, MG) were gathered from the Indian Institute of Technology Guwahati campus (26.1879°N, 91.6916°E) in Assam, India. The collected biomass was rinsed with clean tap water and sun-dried for 24–48 h depending on weather conditions. After sun drying, the sample was placed in a hot air oven at 105 °C for 2 h and then stored in airtight plastic bags to prevent moisture absorption. The dried biomass was ground to a particle size of >1 mm using a lab-grade moisture grinder, as smaller particle sizes improve liquid yield during pyrolysis by enhancing heat and mass transfer.

### 2.2 Physicochemical Characterization of the Marigold Flower

Physical and chemical characteristics were used to describe MG. The first step in characterization is the proximate and ultimate analysis. Assessments of the volatile matter, ash content, and fixed carbon content were made using ASTM D5142 and D1762-84 methods. Further, the elemental composition of biomass and biochar was computed using an elemental analyzer (Euro EA3000 Elemental Analyzer). The heating value is the crucial characteristic that establishes the bioenergy potential of biomass. An oxygen bomb calorimeter (Parr, 6400) was utilized to determine the calorific value of biomass and biochar. The density of biomass has a crucial role in determining the material’s thermochemical or biological processes, as well as in material sizing and fuel storage requirements. With the help of a digital balance and graduated cylinder, the bulk density of the biomass was determined. The complex combination of extractive and hemicellulose, cellulose, and lignin was also estimated by using wet chemistry methods. Hexane and ethanol were used as solvents during the biomass extraction process in a Soxhlet apparatus.

### 2.3 Thermal Analysis

Thermogravimetric analysis (TGA, TG 209 F1 Libra) was used to measure the weight of the sample as a function of temperature or time in a controlled atmosphere. Eight milligrams of the sample was added to the alumina crucible. The studies were carried out in a N_2_ atmosphere at a 10 °C min^–1^ heating rate and a 50 mL min^–1^ purge gas flow from 30 to 800 °C. Under the conditions mentioned above, the kinetic research at dynamic heating rates (10, 30, and 50 °C min^–1^) was carried out in the same TGA. The findings were utilized to compute the kinetic parameters and reaction process.

### 2.4 FTIR Study

The surface functional groups of the material were determined using an FTIR analyzer (IRTracer-100, Shimadzu). The oven-dried sample was combined with KBr at a mixing ratio of 1:100. Further, 128 scans were used to obtain the spectra at 400–4000 cm^–1^ at a resolution of 4 cm^–1^.

### 2.5 Kinetic Analysis

The kinetic analysis is a promising way of unraveling the mechanism of solid-state reactions and estimating the kinetic parameters. Pyrolysis is a complex process; numerous thermal degradation reactions coincide in parallel or in series, so it is impossible to develop a kinetic model that considers all of these reactions. It is usually studied by assuming a first-order pseudo mechanistic reaction model, nonisothermal or isothermal. The following reaction can represent the pyrolysis process of biomass: A_solid_ → B_solid_ + C_volatile_. Model-free methods are applied to calculate the kinetic parameters of biomass. Table 1 lists the models used for kinetic parameters, and Table 2 lists the various models used for biomass decomposition during pyrolysis.

**Table 1:** Kinetic and Thermodynamic Models Are Used to Estimate the Kinetic Parameters^a^^13,14^

nodel name	equation
Kissinger–Akahira–Sunose method (KAS)	(1)
Ozawa–Flynn–Wall (OFW) method	(2)
Friedman method (FM)	(3)
Starink method (STM)	(4)
Criado method	(5); (6)
thermodynamic study	
enthalpy (Δ*H*)	(7)
Gibbs energy (Δ*G*)	(8)
entropy (Δ*S*)	(9)

a *A* is the pre-exponential factor or frequency factor (s^–1^), *T*_m_ is the peak decomposition temperature in K that can be determined by the maximum mass loss rate in the DTG curve, *K*_B_ is the Boltzmann constant 1.381 × 10^–23^ J K^–1^, and *h* is the Plank constant 6.626 × 10^–34^ J s.

**Table 2:** Solid-State Reaction Model for the Decomposition of Biomass during Biomass^15^

mechanism	*f*(*x*)	*g*(*x*)
Avrami-Erofe’ev (A_2_)		
Avrami-Erofe’ev (A_3_)		
Avrami-Erofe’ev (A_4_)		
one-dimensional movement (R_1_)	1	*x*
contracting area (R_2_)		
contracting volume (R_3_)		
one-dimensional diffusion (D_1_)		*x*^2^
two-dimensional diffusion (D_2_)	[ – ln (1 – *x*)]^−1^	(1 – *x*) ln (1 – *x*) + *x*
three-dimensional diffusion (D_3_)		
Ginstling–Brounstein (D_4_)		
first-order (F_1_)	1 – *x*	– ln (1 – *x*)
second-order (F_2_)	(1 – *x*)^2^	(1 – *x*)^−1^ – 1
third-order (F_3_)	(1 – *x*)^3^	((1 – *x*)^−2^ – 1)/2

### 2.6 Pyrolysis Experimental Setup

**Figure 1:**
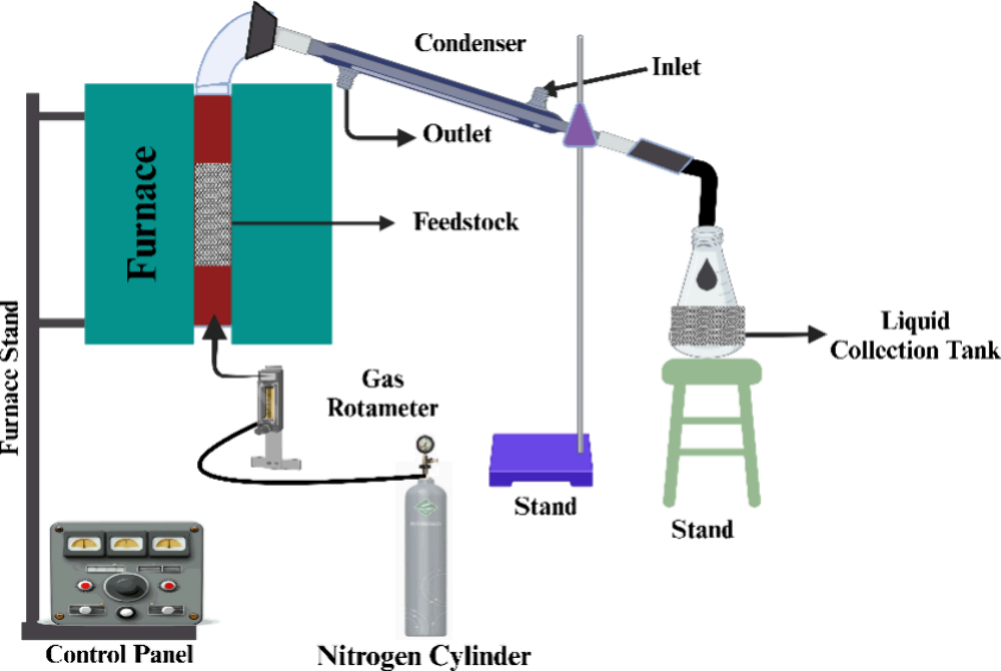
Experimental layout of the pyrolysis setup.

MG was pyrolyzed in an oxygen-free environment using a semibatch fixed bed reactor. With an internal diameter of 4 cm, an external diameter of 4.6 cm, and a length of 30 cm, the reactor was constructed from premium stainless steel (SS-304). To prevent heat loss, the interior surface of the furnace was covered with ceramic bricks, while the exterior was made of premium stainless steel. The main components of the experimental apparatus include a condenser, liquid collection shell, thermocouple, control panel, nitrogen cylindered gas rotameter, and stainless-steel reactors. The reactor was filled (each batch weighing 100 g), and it was positioned vertically within the furnace. A particle size of >1 mm, 450 °C, and a heating rate of 10 °C min^–1^ were used for the pyrolysis test. Assuming minimal heat loss, the furnace was designed to ensure that the heat was raised consistently throughout the reactor. The temperature, residence time, and heating rate were all operated via the control panel directly attached to the furnace. The reactor’s top section was connected to a condenser, while the bottom portion was connected to a nitrogen input. Using a gas rotameter, the nitrogen flow rate was precisely measured and maintained at 100 mL min^–1^ throughout the experiment. Noncondensable gases were released from the condenser, while the hot volatile created was trapped there (condensable gases). After the reactor had cooled to room temperature (30 °C), the biochar was finally removed and placed in an airtight glass container for further study. Figure 1 shows the entire experimental setup for pyrolysis. Equation 10 was used to calculate the yield of the solid.
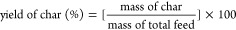
10

### 2.7 Characterization of Biochar

The procedures outlined in Section 2.2 were used to calculate the bulk density, elemental analysis heating value, and proximate study. A Brunauer–Emmett–Teller (BET) surface area analyzer (Micromeritics ASAP-2020 BET) was used to quantify the surface area of biochar while it was adsorbing N_2_. The biochar was heated at a rate of 10 °C min^–1^ for 6 h at 200 °C to be regasified. Additionally, by using ASTM standard D6556-19, the regasified biochar was placed in the BET tube utilizing the adsorption/desorption technique with multipoint BET. The surface morphology of the sample was assessed using EDX analysis and FESEM (FESEM, Zeiss Supra 40) operating between 5 and 15 kV. Using particle size analyzers and a Eutech waterproof (pH Spear) pH meter, the acidity and zeta potential of biochar were determined. Additionally, 1 g of powdered biochar was mixed with 50 mL of distilled water and agitated for 8 h at 25 °C. The water holding capacity (WHC) of marigold flower biochar (MGB) was then determined using deionized water, a filter paper (P8, Fisher brand), and a ceramic Büchner funnel.

## 3 Results and Discussions

### 3.1 Physicochemical Characterization of Biomass

The physicochemical characterization of marigold flower (MG), along with other reported feedstock, such as waste dahlia flowers,^16^*Urochloa mutica*,^17^ and *Miscanthus giganteus*,^18^ is presented in Table 3. The results confirmed that MG has less than 10% moisture (7.87%), making biomass more suitable for pyrolysis. The MG biomass has lower moisture than *Urochloa mutica* and *Miscanthus giganteus* and higher moisture than waste dahlia flowers. Biomass contains more than 10% moisture and requires a separate drying unit, which eventually adds to the cost. A lower moisture content is preferred for the efficient conversion of biomass into products. The net energy available during combustion, gasification, and pyrolysis is reduced by the energy required to eliminate the moisture content.^19^ Further, MG has 77.53% volatile matter (VM), 6.98% ash content (As), and 7.62% fixed carbon (FC). The VM of GM was found to be equivalent to *Miscanthus giganteus*and higher than waste dahlia flowers and *Urochloa mutica*. A larger bio-oil yield is produced due to the higher amount of volatile materials (for fuel production). In addition to the VM, factors like heating rate, reaction time, and temperature are crucial in turning the VM into bio-oil and syngas.^20^ MG’s ash content was higher than *Urochloa mutica* and *Miscanthus giganteus* and lower than waste dahlia flowers. The high ash content in biomass is unwanted because it hurts the combustion and process of gasification and has no positive impact on the energy output.^21^ The ash concentration is also influenced by the planted region’s soil and the irrigation water’s caliber.^21^ The measured fixed carbon content (7.62%) is lower than the quantities most other researchers reported in Table 3. Further, the VM/FC ratio, which gauges the extractable energy content, was found to be 10.17. Our finding was well matched with the value reported for peanut shells.^19^ Further, it was discovered that the contents of carbon, hydrogen, nitrogen, oxygen, and sulfur were found to be 49.23, 6.73, 3.64, 40.39, and 0%, respectively. It was established that biomass has a higher carbon content and provides a higher heating value.^19^ The values of carbon and hydrogen are higher than the values reported in Table 3, which confirm that the energy content of biomass would be higher. Further, the oxygen content is lower than the value reported in Table 3. The lower oxygen is vital for getting high-quality bio-oil because a larger oxygen content reduces the heating value.^19^ MG’s nitrogen content was found to be higher than *Urochloa mutica* and *Miscanthus giganteus* and lower than waste dahlia flowers. Furthermore, the HHV of MG was observed to be 20.76 MJ kg^–1^ because of the elemental difference since biomass’ heating value depends on biomass’ elemental composition. The bulk density of MG was found to be 430.80 kg m^–3^, which is lower than waste dahlia flowers, which eventually confirmed that transportation and storage are a little bit difficult. Biomass is the complex hemicellulose, cellulose, lignin, and extractives matrix. The compositional study of biomass is crucial since each component has its thermal degradation behavior. The biochemical analysis of MG confirmed 23.42, 35.12, and 14.36% to be hemicellulose, cellulose, and lignin, respectively. The cellulose content contributes to the maximum bio-oil during pyrolysis. Lastly, the extractive content of MG is found to be 25.71%, which is very close to the value reported for waste dahlia flowers.^22^ Overall, it was observed that MG can be used as a pyrolysis feed for the production of liquid fuel and chemicals.

**Table 3:** Physiochemical Characterization of MG along with Other Reported Biomass

analysis	MG	waste dahlia flowers^16^	*Urochloa mutica*(17)	*Miscanthus giganteus*(18)
proximate study (wt %) dry basis				
moisture content	7.87 ± 0.16	5.79 ± 0.14	7.23	10.00
volatile content	77.53 ± 1.7	69.50 ± 0.16	68.40	78.80
ash content	6.98 ± 0.05	11.66 ± 0.12	5.0	2.70
fixed carbon	7.62 ± 0.19	13.04 ± 0.11	4.0	9.50
elemental analysis (wt %) dry basis				
C	49.23	44.86	44.73	43.70
H	6.73	5.57	6.88	5.70
S		0.84	0.24	0.20
N	3.64	3.66	0.98	1.10
O	40.39	45.07	46.84	44.80
higher heating value (MJ kg^–1^)	20.76 ± 0.28	16.52 ± 1.2	15.04	17.80
bulk density (kg m^–3^)	430.80 ± 1.5	750 ± 1.6		
total extractives (wt %)	25.71 ± 1.41	28.79 ± 1.2		
hexane	14.48 ± 1.6	18.52 ± 1.8		
ethanol	11.23 ± 0.8	10.27 ± 1.4		
biochemical analysis (wt %)	72.90			
hemicellulose	23.42 ± 0.98	24.22 ± 0.42		
cellulose	35.12 ± 1.52	35.63 ± 0.62		
lignin	14.36 ± 1.09	11.36 ± 0.82		

### 3.2 EDX Analysis of Biomass

An EDX analysis of the biomass reveals a variety of inorganic compounds. Various biomasses have varying concentrations of the mineral content. These substances exist in many forms, such as oxides, carbonates, silicates, sulfates, and phosphates. Comprehending the various minerals present in biomass feedstock is vital, as they may impact the pyrolysis properties, product distribution, and product qualities. The minerals in MG are included in Table 4, and they correspond with those from previous testified investigations. MG has 46.30% K, 9.20% Ca, 6.60% P, 9.90% Si, 12.60% Mg, 2.20% Al, and 4.60% Cl, respectively. It was shown that when biomass was supplemented with K, Ca, P, Si, Mg, Na, Al, Cl, and other mining minerals, the catalytic impact improved the liquid yield of biomass. Also, the introduction of Ca, Al, and Mg enhances the catalytic effect and promotes the production of hot volatiles.^23^ The findings indicated that the mineral compositions of MG, *Delonix regia*, and *Phyllanthus emblica* differ due to various biomass components and geographical locations. To be more precise, the breakdown of lignin in biomass produced more biochar when K is present, but potassium’s actions shift from catalyst to active free carbon site, which is produced by the dissolution of the carbon framework, increasing the rate of char conversion.^24^ Our findings are consistent with previously published research.^25^

**Table 4:** EDX Analysis of Biomass (wt %) Was Matched with Other Testified Literature

mineral matter (wt %)	MG	*Delonix regia*(26)	*Phyllanthus emblica*(26)
P	6.60	1.50	14.40
K	46.30	4.30	25.30
Ca	9.20	1.40	5.10
Mg	12.60	0.50	7.70
Cl	4.60	0.90	4.90
Na		1.00	6.70
Zr	4.80		21.20
Ni		0.30	0.40
Zn		1.00	0.20
Al	2.20	1.00	
Si	9.90		5.40
Cr	0.60	0.20	1.00
Fe	2.50		
Co	0.10	0.30	
Mn		0.50	1.80
Cu	0.60	0.30	1.50
Ti		0.4	

### 3.3 FTIR of Biomass

**Figure 2:**
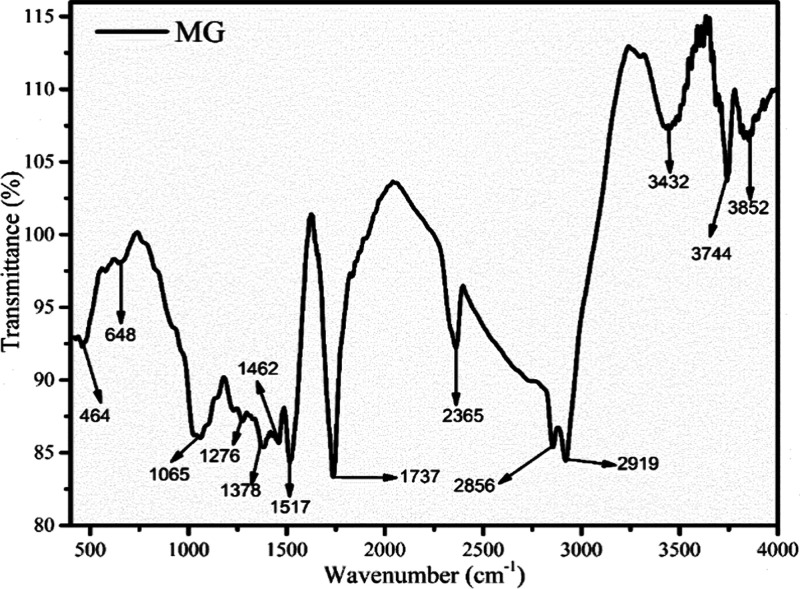
Investigation of the functional group present in MG by using FTIR.

The intricate relationships among the water, phenols, acid, alkane, aliphatic, and aromatic substances were shown by the MG FTIR spectra. A spectrum with wavenumber and transmittance plotted is displayed in Figure 2. To indicate the existence of water, acid, phenols, and aromatic components, an absorption band >3000 cm^–1^ was allotted to the −OH deformation.^27^ The wavelengths of absorption at 2919 and 2856 cm^–1^ were likewise associated with alkane and carbonyl/carboxylic acid and were related to C–H stretching.^28^ The existence of alkyne was demonstrated by peaks in the 1232–1470 cm^–1^ region linked to the C–C bending vibration, but the adsorption band 1517–1737 cm^–1^ related to the C=C stretching vibration made it difficult for aromatics and alkene to survive.^28^ The adsorption band showed the distribution of esters and ether at 1065 cm^–1^, which was linked to the C=O stretching and deformation vibration. The peak region below 1000 cm^–1^ was attributed to O–H bending, demonstrating the dominance of mono- and polycyclic substituted aromatic components.^27^

### 3.4 Thermal Analysis and Effect of Heating Rates

**Figure 3:**
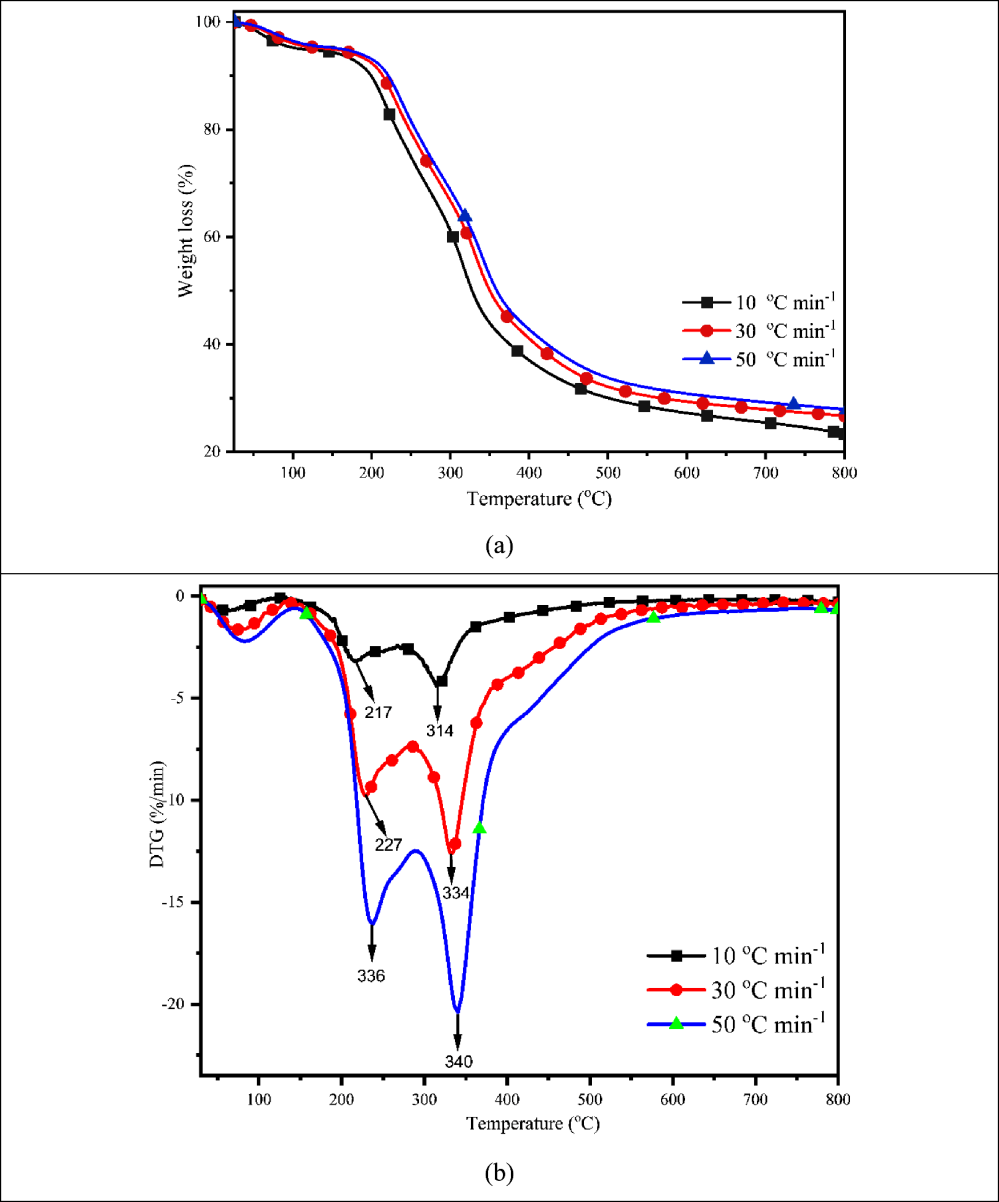
(a) TGA and (b) DTG profiles of MG waste at dynamic heating rates.

The thermal analysis of MG was performed in TGA under nonisothermal situations with a heating rate of 10 °C min^–1^, and the findings are illustrated in Figure 3. Three critical decomposition phases were noted: drying (room temperature to 150 °C), devolatilization (or active pyrolytic stage, 150–500 °C), and biochar production (>500 °C). A similar decomposition profile was observed in *Saccharum munja* biomass.^29^ With a weight loss of 5.63%, a slight hump was seen in the first stage up to 150 °C as a result of the elimination of moisture and low-molecular-weight components. The second stage was thermal degradation, which produced volatiles by breaking down cellulose and hemicellulose at temperatures between 150 and 500 °C and resulted in a total weight loss of 65.98%. The higher-molecular-weight chemical is broken down into smaller-molecular-weight components in this area, which is also referred to as an active pyrolytic zone, by applying constant heat. While cellulose is made up of a long linear chain compound with a d-glucosyl group that has a more ordered structure, hemicellulose is mainly composed of polymerized monosaccharides with a lower degree of polymerization. Because of this, hemicellulose breaks down more quickly and has a lower thermal stability than cellulose, which can also be observed in DTG.^30^ The breakdown of lignin, which resulted in the development of char, was the primary cause of the third stage, also referred to as the passive pyrolytic zone of thermal breakdown, which was detected at 500–800 °C. Lignin degrades at a higher temperature (>500 °C) because of its greater stability due to the incidence of phenolic hydroxyl groups. The derivative of the thermogravimetric analysis (DTG) curve made it clear that the breakdown of hemicellulose occurs around 210 °C, as indicated by the first peak that appears after a slight hump, and the decomposition of cellulose occurs at 330 °C, as indicated by the second peak. During biomass decomposition, lignin does not produce sharp peaks because it decomposes over a broad temperature range due to its complex and heterogeneous structure. This wide range of thermal stability results in a more gradual degradation process, lacking distinct, well-defined peaks.^30^

The effect of heating rate on the thermal behavior of MG was investigated at three distinct heating rates (10, 30, and 50 °C min^–1^) in a TGA under a nonisothermal environment and is shown in Figure 3. It is clear from Figure 3 that when the heating rate escalated, the thermal breakdown curves progressed to a higher temperature zone without impairing the decomposition profile of biomass, increasing the output of volatile compounds and char. The DTG graph shows that, without altering the thermal profile of the breakdown, an increase in heating rates caused the peak to shift to a higher temperature zone (217, 227, and 336 °C for the first peak and 314, 334, and 340 °C for the second peak, respectively) (Figure 3b). Waste dahlia flowers were pyrolyzed in TGA at varying heating rates (5–20 °C min^–1^) in a nonreactive atmosphere by Mishra et al. (2020).^16^ They found that when heating rates increased, TGA curves migrated to the upper-temperature regions (198, 206, and 218 °C, respectively). The pyrolysis properties of sugar cane (*Saccharum officinarum L.*) leaves were investigated in a TGA under nonisothermal conditions using six different heating rates (5 to 40 °C min^–1^).^28^ They found that TGA curves transfer to higher temperature areas as heating rates increase without changing the breakdown patterns. The behavior can be attributed to several factors, including reduced residence time, limited mass transfer, poor heat conductivity, inefficient heat transfer, and altered reaction processes at a higher heating rate. Tar evaporation and char production are two competing reactions that account for the majority of the increase in the total volatile output.^31^ Tar cracking time reduces with increasing heating rate, yielding more tar and less methane.^31^ Because biomass has a low heat conductivity, at the higher heating rate, a temperature gradient would form between the outside surface and the interior core. Thermal lag results from biomass not being heated consistently throughout since there is not enough time for improved heat transfer from the outside surface to the inner core.^32^ The lower heating rates cause volatiles to stay in the reactor longer, which encourage secondary processes that lead to the production of char, such as cracking, repolymerization, and recondensation, as the pace of heating increases, and less volatile substances remain in the air, which prevent secondary reactions and increase yield. The disintegration of MG was found to be 64.38, 62.90, and 61.40%, with an increase in heating rates from 10, 30, and 50 °C min^–1^ at the active zone (about 150–500 °C), respectively. Furthermore, when the heating rate was augmented, the total volume of hot vapors was maximized, but when the heating rate was decreased, the product’s composition altered because cracking, recondensation, and repolymerization may have developed, which would have favored the conversion of biochar.^33^ Volatile constituents at lower heating rates were shown to differ from those at higher heating rates; this difference may have resulted from the volatile products’ shorter residence times at lower heating rates.^33^ Furthermore, the inorganic components at the completion of the trials increased (23.32, 26.68, and 27.83%) with an increase in heating rates from 10 to 30 and 50 °C min^–1^. The shortened residence time lengthened the time of biomass particle contact (partial pyrolysis) and increased the volume of inorganic residue at higher heating rates. The longer residence period increased the interaction between the biomass particles and reduced the quantities of inorganic waste at lower heating rates.^16^

### 3.5 Kinetic Analysis

**Figure 4:**
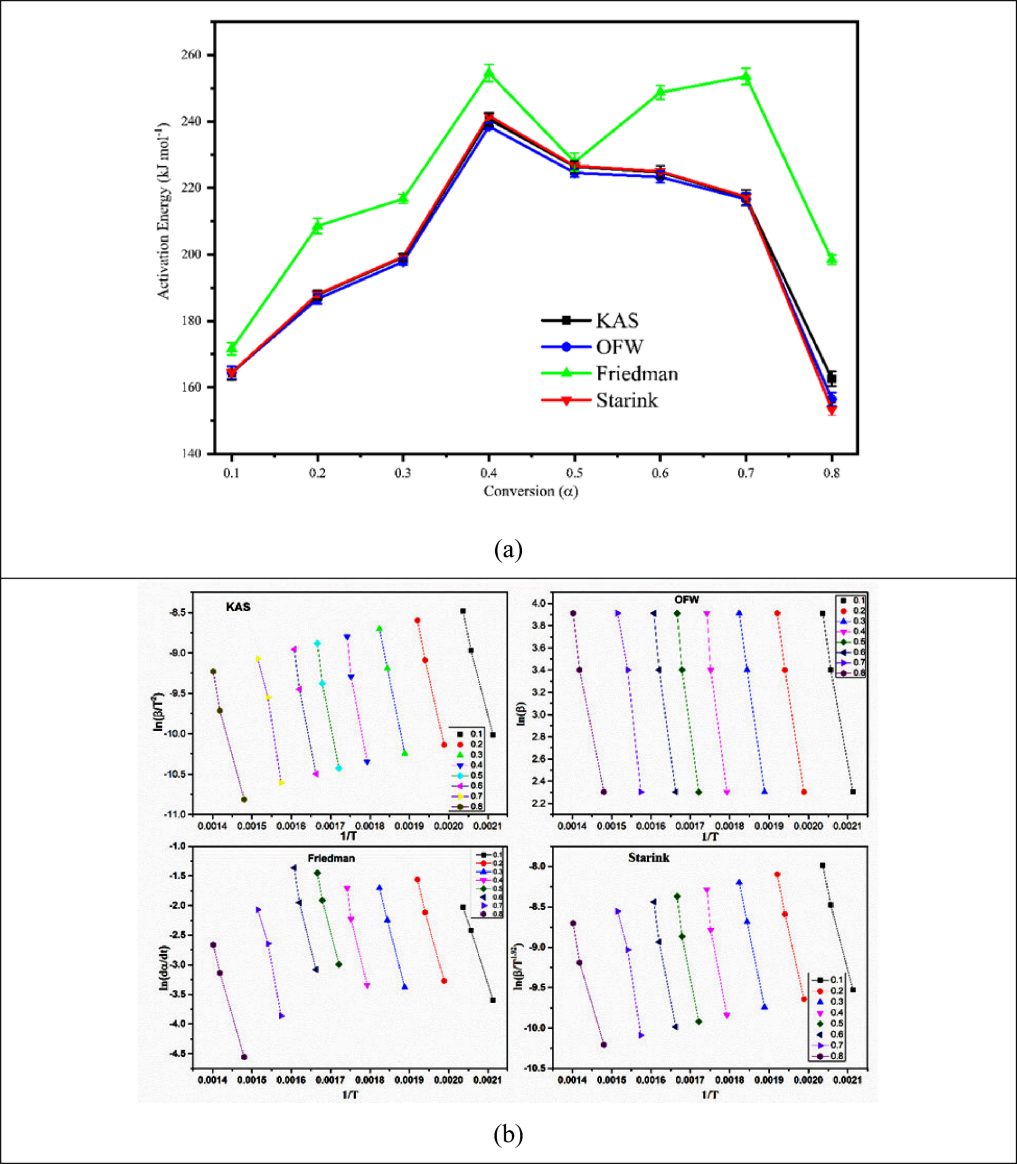
(a) Variation of activation energy against model-free methods with conversion and (b) plotting of various models' fitted data.

Table 5 lists the kinetic parameters of DF biomass that were evaluated at dynamic heating rates of 10, 30, and 50 °C min^–1^ using KAS, FWO, STM, and FM. The conversion value greater than 0.8 was disregarded since the results indicated that the low correlation values made it difficult for it to match the experimental data.^28,34^ Kinetics results show that each conversion value has a correlation value (*R*^2^) of more than 0.95, indicating the most effective fitting value between 10 and 80% conversion. The average activation energies deliberated over the conversion range using KAS, FWO, STM, and FM were found to be 236.35, 232.29, 234.74, and 221.50 kJ mol^–1^, respectively. Table 5 demonstrates that there was a strong association and that the average activation energy determined by the KAS, FWO, and STM approaches was roughly in the same range. However, the FM approach revealed a different estimated activation energy than the others. FM should ideally compute the kinetic parameter precisely since it relies only on the Arrhenius temperature dependence in its kinetic equation and makes no further mathematical approximations. It was more susceptible to experimental noise though because it required instantaneous rate data, and even a small mistake may cause the system to become mathematically unbalanced.^35^ The KAS, OFW, FM, and STM models were employed to figure out the activation energy, which ranged from 164.29 to 291.60, 164.49 to 286.44, 171.61 to 258.42, and 164.46 to 283.40 kJ mol^–1^, respectively. The activation energy fluctuated with the conversion value since hemicellulose, cellulose, and lignin compose the majority of the biomass. The findings demonstrated that activation energy fluctuated with conversion value, suggesting that many reaction stages were necessary for the fragmentation and conversion of biomass rather than a single step. Additionally, it was discovered that the average frequency factors for KAS, OFW, FM, and STM were found to be 4.00 × 10^21^, 1.44 × 10^21^, 5.14 × 10^21^, and 2.65 × 10^21^ min^–1^, respectively. It was also seen that the pre-exponential factor altered the conversion value, indicating that the biomass advanced through a sophisticated reaction mechanism.^36^Figure 4a shows the fluctuation of activation energy concerning the conversion value, and Figure 4b indicates the curve fitting. The breakdown of the various components of biomass led to an apparent activation energy gain of up to 45% conversion and a subsequent decline. It was also observed that the activation energy provided by each model varied; this variation might have resulted from the use of various approximation techniques when solving the models mathematically.^37^ Nevertheless, it was also discovered that the pyrolysis reaction followed several reaction steps rather than just one. Nonetheless, the makeup of the sample, the kind of model employed, the experimental setup, and the mathematical computations were discovered to be responsible for the slight variance in the activation energy.

**Table 5:** Kinetic Analysis of Marigold Flowers (MG) Using Different Model-Free Methods

α	KAS method			Starink method			OFW method			Friedman method		
	*R*^2^	*E* (kJ mol^–1^)	*A* (s^–1^)	*R*^2^	*E* (kJ mol^–1^)	*A* (s^–1^)	*R*^2^	*E* (kJ mol^–1^)	*A* (s^–1^)	*R*^2^	*E* (kJ mol^–1^)	*A* (s^–1^)
0.1	0.9965	164.29	1.18 × 10^18^	0.9965	164.46	1.37 × 10^17^	0.9962	164.49	1.36 × 10^17^	0.9996	171.61	2.67 × 10^17^
0.2	0.9988	187.84	2.86 × 10^19^	0.9988	188.02	7.00 × 10^18^	0.9989	186.63	5.03 × 10^18^	0.9983	208.56	2.15 × 10^20^
0.3	0.9999	199.18	3.78 × 10^19^	0.9999	199.38	1.48 × 10^19^	0.9999	197.84	1.04 × 10^19^	0.9998	210.74	1.15 × 10^20^
0.4	0.9822	240.69	3.17 × 10^22^	0.9813	241.73	2.11 × 10^22^	0.9825	238.54	1.14 × 10^22^	0.9817	214.59	4.02 × 10^22^
0.5	0.9891	256.42	1.80 × 10^20^	0.9891	258.63	1.36 × 10^20^	0.9899	244.55	9.03 × 10^19^	0.9933	227.79	2.99 × 10^19^
0.6	0.9915	269.75	2.44 × 10^19^	0.9916	264.97	2.45 × 10^19^	0.9922	263.30	1.75 × 10^19^	0.9867	236.72	4.50 × 10^20^
0.7	0.9788	281.07	4.83 × 10^17^	0.9789	277.33	6.41 × 10^17^	0.9808	276.58	5.47 × 10^17^	0.9815	243.57	5.27 × 10^19^
0.8	0.9898	291.60	1.44 × 10^12^	0.9857	283.40	4.96 × 10^11^	0.9875	286.44	9.21 × 10^11^	0.9984	258.42	1.14 × 10^14^
Avg.		236.35	4.00 × 10^21^		234.74	2.65 × 10^21^		232.29	1.44 × 10^21^		221.50	5.14 × 10^21^

### 3.6 Master Plot

**Figure 5:**
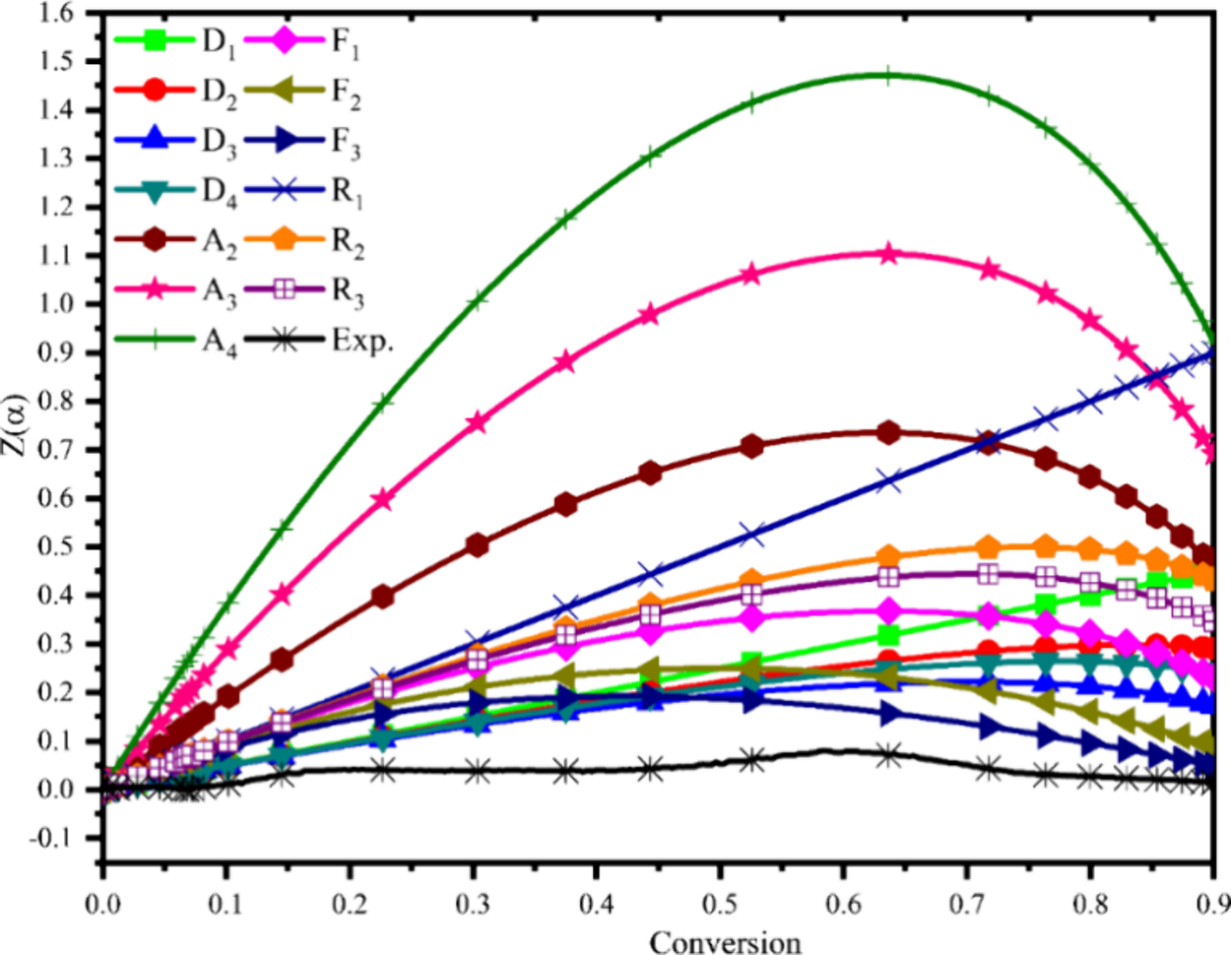
Master curve and experimental curve of MG obtained by using the Criado method.

Understanding the conversion rate, activation energy, and pre-exponential factor is essential for building biomass pyrolyzers. The Kinetics Committee of the International Confederation for Thermal Analysis Calorimetry (ICTAC) authorized several iso-conversional model-free kinetic techniques, including the linear regression method, to determine these three kinetic parameters for each heating rate.^38^ The thermal breakdown mechanism reported by Criado et al. (1989) was estimated using the average activation energy for MG found from OFW and is presented in Figure 5.^12^ The solid state allowed for an endless number of series and parallel reactions, which meant that the reaction mechanism was intricate multistep rather than simple single-step. The theoretical reference curve, termed a master curve, is computed using eqs 5 and 6 and is a derivative of the *f*(*x*) and *g*(*x*) functions shown in Table 1. The breakdown of the solid reaction mechanism is determined by comparing these theoretical curves with experimental data.^12^ The algebraic equation has been divided into five major sets, denoted as Fn, Dn Rn, An, and Pn, respectively (Table 2). These mechanisms include the random breakdown of nuclei, the diffusion process related to heat transfer capacity, the reaction mechanism determined by the material’s surface, and the nucleus formation procedures for the prorogation of thermal deprivation. *Z*(*x*) was determined by using the activation energy that was derived from the OFW model and the heating rate of 10 °C min^–1^. The trend of variation may be associated with the various ways that hemicellulose, cellulose, and lignin interact. Endothermic processes were indicated by a rise in activation energy from 0.1 to 0.6, while exothermic reactions were indicated by a reduction in activation energy beyond 0.6. The cause of this variation in activation energy was the breakdown of bonds. Weak bonding and the depletion of volatile components initially led to lower activation energy. A low activation energy was noted as a result of certain weak bonds breaking and the early elimination of lower-molecular-weight constituents. As the pyrolysis process developed, stronger bond dissociation needed more activation energy. Activation energy dropped as conversion increased because, at high conversion, a lower energy barrier was needed for breakdown. Pre-exponential values varied with conversion, which can be attributed to the complicated interactions between the various components of a biomass sample as well as the complex processes that occur during decay. The experimental results and the master curve are shown in Figure 5. From Figure 5, MG was examined in the conversion value range of 0.1–0.9. The data verified the overlap of the D_1_, D_2_, and D_3_ curves. The decay mechanism in a diffusion approach takes place in single, double, or triple dimensions.^12^ When compared to previous studies, the characteristics of our results are pretty comparable.^28^ The outcomes demonstrated that diffusion occurred throughout the sample at a lower conversion value. Additionally, it was noted that biomass tends toward random nucleation at conversion values greater than 0.5 (F1 denotes unplanned nucleation with a single nucleus in a distinct particle).^12^ This mechanism of reaction serves as a growth center for breakdown reactions and is initiated when degradation is injected from random positions. The temperatures above the 0.6 conversion value cause biomass to split at 350 °C and encourage the disintegration of cellulose chains. Lower-molecular-mass networks are anticipated to serve as the breakdown reaction’s centers of random nucleation and growth. Furthermore, the *Z*(α) value overlaying the D_3_ reaction was detected, indicating a three-dimensional diffusion activity. This might be explained by the hemicellulose and extractives breaking down at a lower temperature. Furthermore, this phenomenon might also be explained by the hot gases that are dispersed around the sample, speeding up the fragmentation of cellulose in addition to diffusion.

### 3.7 Thermodynamic Analysis

The thermodynamic analysis of castor seed and marigold flower is shown in Table 6. The thermodynamic parameters Δ*H*, Δ*G*, and Δ*S* were assessed at *T* = *T*_m_ (*T*_m_ is the DTG peak temperature). Enthalpy reflects the energy differences between the reactant and the activated complex; it can indicate the nature of pyrolysis. A positive value of enthalpies Δ*H* signifies that MG biomass needed more energy to decompose and degradation reactions to be endothermic. A slight difference (∼5 kJ mol^–1^) was observed between the activation energy (*E*) and enthalpy Δ*H* at each conversion point. This variation reflects the favorability of product formation due to the lower potential energy barrier. Moreover, it shows that the formation of the product would be easier to achieve. The degree of disorder in the product resulting from bond dissociation concerning the original reactant is represented by entropy. In contrast to negative values, positive values of Δ*S* show that the activated complex formed by bond dissociations has more disorder than the initial reactants. The system’s entropy change also indicates how close it is to reaching thermodynamic equilibrium. The lower Δ*S* revealed a state that had recently experienced some process and was close to its thermodynamic equilibrium. Reactants, in this instance, exhibit minimal reactivity. As a result, it takes longer for the active complex to develop. On the other hand, the higher Δ*S* reflected high reactivity, and the system can react faster to produce the activated complex. MG has a positive entropy (76 to 51 kJ mol^–1^.K^–1^), which shows a more activated and disorder-activated complex. Δ*G* indicates the degree and spontaneity of the reaction. In addition, it reflects the internal energy that is provided by biomass during pyrolysis. Marigold flowers (MG) have average Δ*G* values of 159–171 kJ mol^–1^, which were observed to be higher than para-grass (168–173 kJ mol^–1^),^17^ rice straw (164.59 kJ mol^–1^), dairy manure (165.086 kJ mol^–1^), rice bran (141.62 kJ mol^–1^), and chicken manure (158.90 kJ mol^–1^).^39^

**Table 6:** Thermodynamic Analysis of MG using KAS, STM, OFW, and FM Models^a^

α	KAS method			Starink method			OFW method			Friedman method		
	Δ*H*	Δ*G*	Δ*S*	Δ*H*	Δ*G*	Δ*S*	Δ*H*	Δ*G*	Δ*S*	Δ*H*	Δ*G*	Δ*S*
0.1	159.23	127.13	52.79	159.41	138.21	34.87	159.43	138.24	34.85	166.56	141.98	40.42
0.2	182.79	134.57	79.29	182.97	141.89	67.57	181.58	142.16	64.83	203.51	145.10	96.06
0.3	194.13	144.51	81.60	194.32	149.47	73.77	192.78	149.66	70.92	211.69	156.41	90.91
0.4	235.64	151.99	137.57	236.68	155.09	134.18	233.49	154.99	129.10	249.53	164.68	139.55
0.5	221.36	163.86	94.58	221.57	195.81	42.37	219.50	165.48	88.84	222.74	174.30	79.65
0.6	219.69	172.29	77.96	219.92	172.51	77.98	218.24	172.50	75.22	243.67	181.53	102.20
0.7	212.02	184.45	45.34	212.28	183.28	47.69	211.52	183.32	46.38	248.52	197.22	84.37
0.8	157.55	194.27	–60.40	148.34	190.48	–69.29	151.39	190.39	–64.14	193.36	207.99	–24.06
avg.	197.80	159.13	63.59	196.94	165.84	51.14	195.99	162.09	55.75	217.45	171.15	76.14

a Note: Δ*H* and Δ*G* are in kJ mol^–1^, and Δ*S* is in J mol^–1^ K^–1^.

### 3.8 Characterization of Biochar

#### 3.8.1 Physicochemical Study of Biochar

The marigold flower-derived biochar (MGB) was collected upon cooling the pyrolysis reactor to room temperature and subjected to physical and chemical analysis. Table 7 shows that the physicochemical properties of MGB are similar to those of *Samanea saman* seed biochar,^6^ coal, palm shell hydro char (PSHC),^40^*Miscanthus* biochar,^41^ brewer’s spent grain,^42^ and ramie residue char.^43^ While grape marc pyrolysis produced 65.80 and 59.10% biochar at 300 and 400 °C, respectively, thermal pyrolysis of MGB produced 36.64 wt % biochar, 29.67% liquid fuel, and 33.69% syngas, respectively.^44^ The proximate analysis demonstrated that MGB had 7.45% moisture, 27.31% volatile matter, 20.04% ash content, and 45.20% fixed carbon. Reduced moisture and increased volatile matter indicated that the biochar ignited more easily during combustion. Furthermore, the absence of moisture suggested that biochar may be kept for a long time and used for a variety of purposes.^40^ An increased ash level (20.04%) was detected, indicating unfavorable outcomes when solid fuel was used. Because of the likely differences in the feedstocks, the moisture content of MGB was found to be higher (7.45%) than that of grape marc biochar (3.4–4.5), *Samanea saman* seed biochar, brewer’s spent grain, coal, and palm shell hydro char. The ash content (20.04%) was found to be greater than that of grape marc biochar (4.9–8.5%), *Samanea saman* seed biochar, brewer’s spent grain, coal, and palm shell hydrochar. Furthermore, the increased heating value was decreased by biochar enhanced with a higher ash content. Finally, the amount of fixed carbon was found to be 45.20%, which is lower than the value reported for other biomasses in Table 7. Fixed carbon is a critical component in fuels like coal and biomass, as it represents the carbon remaining after volatile matter has been expelled. It is crucial for assessing the fuel’s energy content and combustion efficiency. A higher fixed carbon content generally indicates a higher energy yield and longer burn time, making it an essential parameter in evaluating fuel quality.^45^ Elemental analysis of MGB revealed the presence of 57.10% carbon, 2.54% hydrogen, 36.56% oxygen, and 3.79% nitrogen; the sulfur content was missing. There is substantial agreement in the elemental analysis of biochar with grape marc at 500 and 600 °C^44^ but lower than brewer’s spent grain biochar at 400 and 500 °C. It has been demonstrated that biochar’s nitrogen content increased its suitability for soil application.^46^ MGB’s reported nitrogen levels of 3.79% are in line with that of biochar generated from grape marc at 600 °C but lower than the biochar derived from brewer’s spent grain biochar at 400 and 500 °C. When employed as a soil enhancer, a higher percentage of carbon (57.10%) and oxygen (36.56%) subsequently became a supply of nutrients for plants.^46^ Moreover, oxygen-enriched biochar significantly improves its characteristics during the leaching process. The biochar carbon content is substantial because it reflects the material’s stability and long-term carbon sequestration potential, as well as its ability to improve soil fertility by enhancing soil structure and nutrient retention. However, hydrogen content is indicative of the biochar’s volatile matter and reactivity, influencing its effectiveness as a soil amendment and its performance in environmental applications such as water filtration and pollution remediation.^45^ The molar ratio of biochar showed excellent solid fuel qualities. The higher O/C ratio (0.28), which indicates a lower energy content, is regarded as detrimental, whereas the higher H/C ratio (0.56), which indicates a higher energy content, supports a good fuel potential based on the elemental composition of biochar. MGB was observed to interact with an O/C ratio lower than that of activated carbon (0.10). Further, MGB has a greater H/C ratio than other verified biochar, which qualifies it as the primary boiler fuel feed. Additionally, the ratio of oxygen/carbon denotes the polarity and richness of the polar oxygen-containing surface functional groups in the biochar. In contrast, the H/C ratio shows the stability and aromatic content of the biochar. In comparison to the biochar given in Table 7, the HHV of the MGB was discovered to be 33.57 MJ kg^–1^. Additionally, biochar has an excellent HHV and is a solid fuel that may be used in heating and cooking sectors. Because of the removal of oxygen from the biochar, the carbon content of MGB was found to be larger (57.10%) than that of raw biomass (40.39%).^40^ Furthermore, MGB contains 3.79% more nitrogen than fresh biomass (3.64%), which is a result of several chemical reactions that occur during pyrolysis, including moisture exclusion, decarbonylation, and decarboxylation.^40^ Biochar is a substance that is widely recognized for treating water and wastewater.^47^ A decreased BET surface area (9.96 m^2^ g^–1^) was found during the MGB characterization, which limited its usefulness as a bio-adsorbent. The BET surface area is not the sole criterion for evaluating the bio-adsorbent capability of biochar. When choosing bio-adsorbents, the Zeta potential of the biochar is also crucial. With a Zeta potential of −29.60 mV (millivolt), which is comparable to that of activated carbon (−29.14 mV), MGB was shown to be a possible adsorption choice for wastewater and water treatment.^40^ The MGB has been determined to have an alkaline pH of 7.61 and can be used as a soil enhancer.^40^ The acidity of the MGB was found to be very close to the value reported for brewer’s spent grain biochar at 400 and 500 °C. Activated carbon has a bulk density of 221 kg m^–3^, whereas MGB bulk density was found to be 203.40 kg m^–3^, suggesting that biochar may be stored and transported with ease. Further, the water holding capacity (WHC) of MGB was found to be 39.71%, which is higher than the brewer’s spent grain biochar and suitable for soil enhancers. The water-holding capacity (WHC) of biochar is critical for enhancing soil moisture retention, improving water availability for plants, and mitigating drought stress. It supports sustainable agriculture by reducing irrigation needs, promoting soil structure, and fostering microbial activity, thus contributing to improved crop yields and long-term soil health.^48^

**Table 7:** Physicochemical Characterization of MGB and Compared with Other Reported Biochar and Coal

analysis	MGB	*Miscanthus* biochar^41^	*Samanea saman* seeds biochar^6^	coal^40^	palm shell hydro char^40^	ramie residue char^43^	brewer’s spent grain (400 °C)^42^	brewer’s spent grain (500 °C)^42^
	Proximate analysis (dry basis, wt %)							
moisture	7.45 ± 0.24	2.45 ± 0.04	5.14				4.66 ± 0.69	3.46 ± 0.26
volatile matter	27.31 ± 0.44	34.26 ± 0.025	34.14			16.91	5.83 ± 0.69	49.27 ± 2.02
ash content	20.04 ± 0.36	12.25 ± 0.68	13.18			7.54	8.42 ± 0.65	8.59 ± 1.22
fixed carbon	45.20 ± 0.26	51.97 ± 1.1	47.54			75.55	38.75 ± 2.0	42.14 ± 1.22
	Elemental analysis (dry basis, wt %)							
C	57.10	70.61 ± 0.2	62.66	55.38	63.77	79.31	62.92 ± 2.11	64.51 ± 5.29
H	2.543	2.27 ± 0.1	2.06	5.86	4.40	2.52	4.30 ± 0.14	4.09 ± 0.26
O	36.56	25.38 ± 0.2	31.83	34.07	23.33	10.45	24.58 ± 0.35	22.93 ± 1.03
N	3.79	1.74 ± 0.1	3.45	2.48	0.52	0.15	8.19 ± 0.29	8.46 ± 0.61
S				2.21	1.02	0.03		
O/C	0.27	0.28	0.38			0.13		
H/C	0.56	1.02	0.40			0.03		
higher heating value (MJ/kg)	33.57 ± 1.89	30.65 ± 1.1	23.14	22.54	26.80	28.40		
BET surface area (m^2^/g)	9.96 ± 0.23	5.50 ± 0.8	8.20		12.26	27.74		
bulk density (kg/m^3^)	203.40 ± 0.82	257 ± 1.2	478					
acidity (pH)	7.61 ± 0.20	7.87 ± 1.1	7.60				7.15 ± 0.12	6.97 ± 0.33
zeta potential (mV)	29.14 ± 0.63	–29.60 ± 1.6						
water holding capacity (%)	39.71 ± 1.21	38.71 ± 0.8					16.16 ± 0.60	15.32 ± 0.08

#### 3.8.2 FTIR Analysis of Biochar

**Figure 6:**
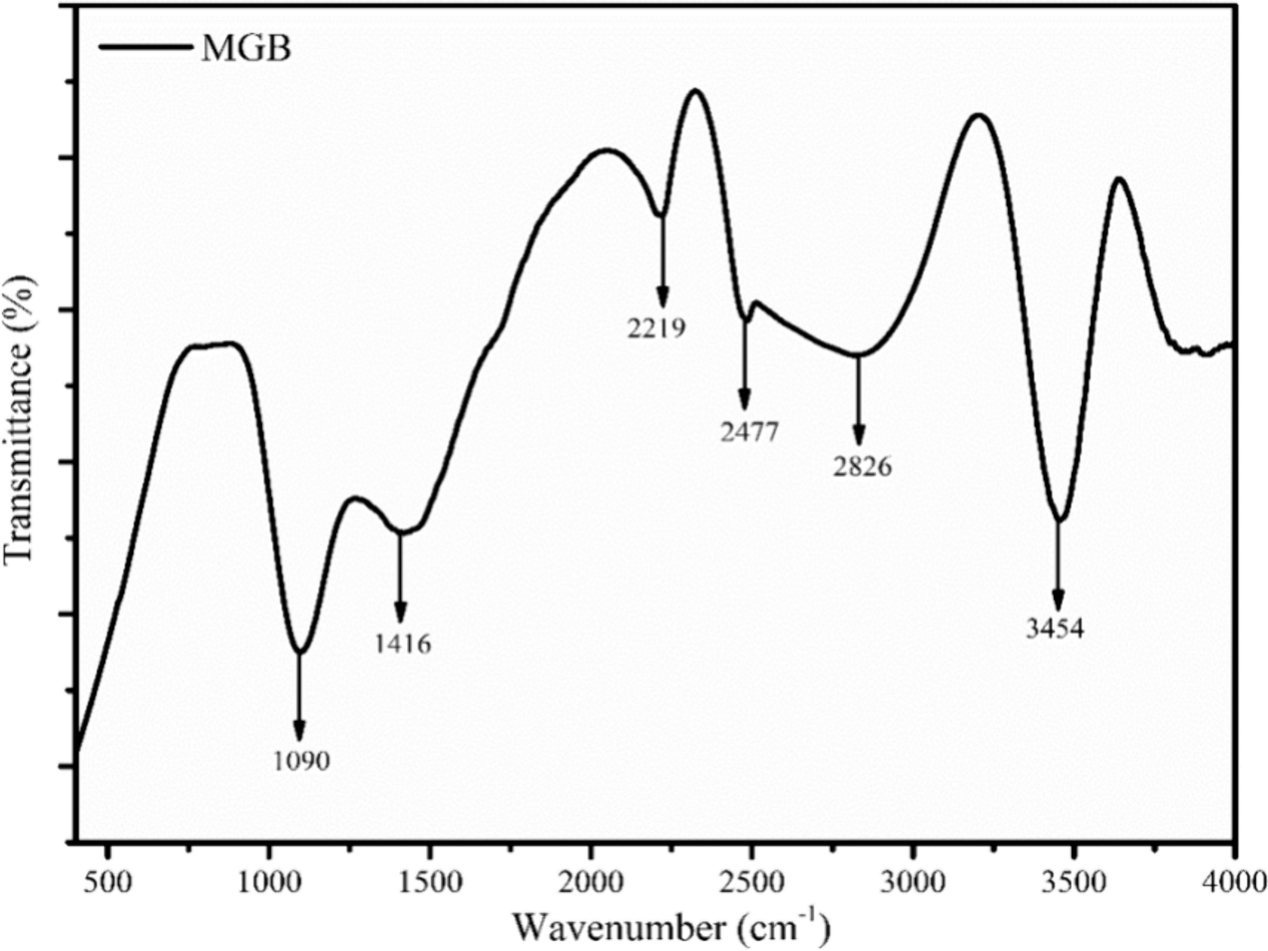
FTIR study of char obtained from the pyrolysis of MGB.

Figure 6 presents the MGB functional group analysis against the transmittance and wavenumber. The C–H bending vibration was attributed to the hydrocarbon (alkane) and aromatics at band peak 1416 cm^–1^.^49^ Furthermore, the C–O stretching vibration at peak 1090 cm^–1^ indicates that MGB is abundant in phenolic compounds. The presence of aromatics in biochar was principally indicated by the band peak of 2477 cm^–1^ associated with C–C stretching vibrations.^50^ The band peak showed the stretching vibration of chemically attached hydroxyl groups at 3450 cm^–1^, which also proved the chemically bound organic OH groups. Alkynes were present in the band peak region spanning 2826–2219 cm^–1^, which were caused by the C–C stretching vibration.^50^ It was discovered that biochar was enhanced with a variety of functional groups, including alkanes, aromatics, hydroxyl groups (moisture), and phenolic substances.

#### 3.8.3 FESEM and EDX Analysis of Biochar

**Figure 7:**
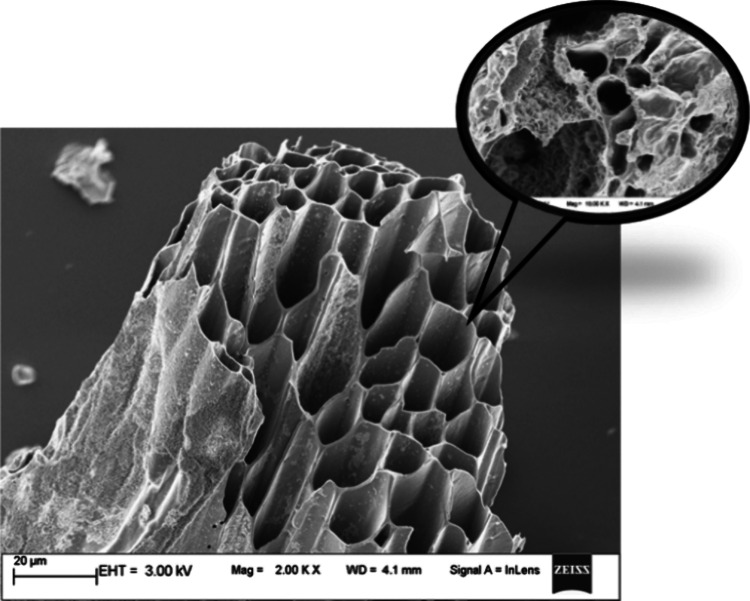
FESEM image of biochar obtained from the pyrolysis of MG.

The MGB surface morphology investigation was conducted using an FE-SEM analyzer, and the pictures are displayed in Figure 7 at magnifications of 2.00 kx. The surface morphology of the MGB showed an extremely complicated structure because various types of minerals were aggregated. In a matter of seconds, pyrolysis causes several processes that change the structure of MGB, including dehydration, decarbonylation, and decarboxylation.^51^ As a result of the breakdown and volatilization of raw materials during pyrolysis, MGB was shown to have a limited number of pores in Figure 7.^52^ A variety of reactions take place over short times during pyrolysis, allowing different volatiles to enter the biochar’s pores and change the particle surface by shrinking, splitting, and other processes. The surface structure of MGB was found to be rough and regular overall, with a few lengthy channels that were created when chemical bonds were broken during pyrolysis. Additionally, the mineral content present in biochar was estimated using EDX analysis and is presented in Table 8. It provides knowledge of the nutritional characteristics of biochar and their impact on crop yield and production directly and indirectly. The composition of the feedstock, the temperature at which pyrolysis occurs, and the accessibility of nutrients during pyrolysis are some of the variables that can affect the nutritional qualities of biochar. All of the biochar’s components, like Na, K, P, and Ca, contributed to soil amendment and improved plant development.^53^ Additional soil micronutrients, such as Si, Al, Fe, and Mg, were also discovered in varying amounts. Biochar Wildey replaced the use of chemical fertilizers and reduced the environmental impact and soill fertility.^53^

**Table 8:** EDX Analysis of the MGB

mineral matter	MGB (wt %)
P	6.8
K	49.4
Ca	10.6
Mg	4.4
Cl	5.4
Na	0.8
Zr	3.1
Ni	0.8
Al	2.0
Si	2.5
Cr	0.2
Fe	1.1
Mn	0.3
Cu	1.7
F	10.9

## 4 Possible Application of Biochar

Biochar is a versatile material with diverse applications in agriculture, environmental remediation, energy storage, industry, and cosmetics. In agriculture, biochar is primarily used as a soil amendment to enhance soil fertility, water retention, and microbial activity. It improves nutrient availability, reduces fertilizer leaching, and stabilizes soil pH, promoting sustainable farming practices. Additionally, biochar sequesters carbon in soil, playing a vital role in climate change mitigation by reducing greenhouse gas emissions. Its high porosity and surface area make it a valuable material for energy storage applications, such as supercapacitors and batteries. When modified or activated, biochar demonstrates an enhanced electrical conductivity, enabling its use in advanced energy technologies. In the metallurgy industry, biochar serves as a renewable substitute for traditional coke or coal, particularly in smelting processes.^54^ Its high carbon content and lower environmental impact contribute to cleaner and more sustainable metal production. Biochar’s exceptional adsorption properties are widely utilized in environmental applications, including the removal of heavy metals, organic pollutants, and dyes from water and air.^55^ This makes it an effective tool for wastewater treatment and environmental cleanup efforts. As a filler in polymer composites, biochar enhances mechanical strength, thermal stability, and biodegradability, providing an eco-friendly alternative to synthetic fillers in materials development.^56^ In cosmetics, biochar is used in products such as facial masks and scrubs for its detoxifying and exfoliating properties. Its porous structure helps absorb oils and impurities, making it a sought-after ingredient in skincare formulations.^57^ Furthermore, biochar is gaining attention as a catalyst and catalyst support in chemical reactions, including biomass conversion and environmental catalysis.^58^ Its tunable surface chemistry and structural stability enable applications such as hydrogen production, biodiesel synthesis, and pollutant degradation.^58^ The wide-ranging applications of biochar across industries highlight its potential as a sustainable solution to address environmental challenges and promote innovation in various sectors.

## 5 Conclusions

The present study analyzed the pyrolysis behavior of waste flowers, providing insights into kinetic parameters and biochar production. The thermogravimetric analysis revealed a multistage decomposition process primarily driven by the breakdown of hemicellulose, cellulose, and lignin at distinct temperature ranges. The activation energy calculated through model-free methods (KAS, OFW, STM, and FM) indicated that the pyrolysis kinetics was significantly influenced by the organic composition of the feedstock, with moderate energy requirements favoring a sustainable conversion process. The biochar produced at 450 °C demonstrated enhanced physicochemical properties, including higher carbon content (57.10%), significant surface area (9.96 m^2^ g^–1^), and favorable functional groups (C–H, C=H, C–C, etc.), making it a promising candidate for applications in soil amendment, pollutant adsorption, and energy storage. The FESEM results confirmed a rough and irregular surface structure. Overall, the pyrolysis of MG not only offers a viable route for managing organic waste but also contributes to sustainable resource recovery by converting waste into value-added products.
